# Ipsilateral femoral neck and trochanter fracture

**DOI:** 10.4103/0019-5413.80052

**Published:** 2011

**Authors:** A Raviraj, Ashish Anand

**Affiliations:** *Department of Orthopaedic Surgery, Fortis Hospitals Ltd., Bannerghatta Road, Bangalore, India*

Sir,

We read the article titled “Ipsilateral femoral neck and trochanter fracture” by Neogi *et al*.^1^ with interest. We have the following concerns on this report. The presence of an ipsilateral fracture neck of femur (NOF) and trochanter is rare and seen in elderly osteoporotic patients due to low energy injuries. However, the occurrence of such injuries in young patients due to high energy injury is also published in the literature.^2^,^3^

There is lack of clarity as to the surgical approach, the authors used to fix the acetabulum and ipsilateral shaft of femur on the right side. We are of the opinion that the acetabular fracture could have been fixed through the posterior approach, without a trochanteric osteotomy. The ipsilateral femoral shaft fracture has been fixed with extensive exposure which amounts to blood loss and soft tissue injury in a polytrauma patient. This could have been avoided if the authors had used an intramedullary interlocking nailing once the acetabulum is fixed, for obvious advantages. The authors describe putting the patient on a fracture table once the right-sided fractures were fixed. The authors fail to describe the technique used to reduce the fracture NOF and trochanter fracture on the left side. Whether the traction was used to reduce the fracture? In young patients, the fracture NOF takes priority over the trochanteric fracture. No less than absolute reduction of the fracture NOF should be accepted. The authors have accepted a varus reduction for fixation of the proximal femoral fractures. This is not biomechanically acceptable as the tensile forces cannot be converted to compressive forces across the fracture site.

Hypothetically, if on the fracture table a good reduction was obtained after traction, this particular fracture could have been fixed with a third generation nail such as reconstruction nail/proximal femoral nail antirotation (PFNA) with minimal soft tissue injury. This could have avoided an extensive dissection in a polytrauma patient, thus reducing the blood loss and surgical time. The intramedullary implant would be superior biomechanically with it being a load sharing device, unlike the dynamic condylar screw (DCS) used by authors which is a load sparing device.^4^

If they had not got a good reduction on the fracture table, the authors should have performed an open reduction of the fracture NOF and the trochanter to get a good reduction and the normal neck shaft angle. In such a scenario, the dynamic hip screw (DHS) with antirotation screw could have been used in maintaining the normal neck shaft angle. We fail to understand why a varus reduction was accepted and DCS was used to fix a fracture where achieving the normal neck shaft angle was crucial to prevent complications. The DCS screw is placed in the inferior head and is found to cut out through the inferior neck cortex in the follow-up x-rays shown in the report. The authors fail to describe the technique of reduction and fixation of the trochanter and NOF fracture, which should be the true focus of this report.

In contrast to what the authors have described, in a polytrauma patient, more minimally invasive fixation surgeries with less blood loss, with shorter operative time and minimal soft tissue injury, should have been considered.

There is no mention of deep vein thrombosis prophylaxis for the polytrauma patient.
